# Chest CT manifestations with emphasis on the role of CT scoring and serum ferritin/lactate dehydrogenase in prognosis of coronavirus disease 2019 (COVID-19)

**DOI:** 10.1186/s43055-021-00459-4

**Published:** 2021-03-30

**Authors:** Rehab Abdel Rahman El Bakry, Ayman Ibrahim Tharwat Sayed

**Affiliations:** 1 Department of Radiodiagnosis, Faculty of Medicine, Ain Shams University, Cairo, Egypt; 2 Department of Anesthesia, Intensive Care and Pain Management, Faculty of Medicine, Ain Shams University, Cairo, Egypt

**Keywords:** Coronavirus, COVID-19, Chest CT, Ferritin, LDH

## Abstract

**Background:**

In March 2020, the World Health Organization announced coronavirus disease 2019 (COVID-19) a pandemic, and because of the primary pulmonary manifestations of the disease, chest CT is essential in the evaluation of those patients. The aim of the study was to evaluate the role of chest CT findings and chest CT scoring along with serum ferritin and LDH in the prognosis of COVID-19 patients in a cohort of the Egyptian population.

**Results:**

This retrospective study included 250 patients with positive RT-PCR for COVID-19, 138 males [55.2%] and 112 females [44.8%], age range 17–82 years with median 49.5. Two hundred patients had a positive significant correlation between age, serum ferritin, serum LDH, and CT score. Bilateral affection was 88% while unilaterality was 12%, and peripheral chest CT findings were stratified as follows: mild [score from 1 to 10], 114 patients [57%]; moderate [score from 11 to 19], 65 patients [32.5%]; and severe [score from 20 to 25], 21 patients [10.5%]. In severe cases, males constitute 85.7% while females were only 14.3%. Statistical and central distribution was 67%, peripheral was 31%, and central was 2%. Ground glass opacity (GGO) was the highest pattern 39.2%, consolidation 31.2%, fibrosis 15.2%, and CP 13.7%, with lymph nodes only 0.6%. Fifteen cases [6%] were critical; all showed severe scores ranging from 21 to 23 with three times increase in serum ferritin and four times increase in LDH. A follow-up study done to 8 cases [3.2%] showed an increase in CT scoring, serum ferritin, and serum LDH.

**Conclusion:**

Chest CT findings are crucial for early diagnosis of COVID-19 disease especially for asymptomatic patients with old age and male sex considered risk factors for poor prognosis. Chest CT score, serum ferritin, and serum LDH help in predicting the short-term outcome of the patients aiming to decrease both morbidity and mortality.

## Background

A viral pneumonia of unknown etiology appeared in China in late December 2019, and the Chinese scientific centers declared that the cause is a new strain of coronavirus named severe acute respiratory syndrome coronavirus 2 (SARS-CoV-2), and the disease is named coronavirus disease 2019 (COVID-19) [1]. It is a highly contagious disease, and by March 2020, the World Health Organization declared it as a pandemic causing a global health emergency [2] making early diagnosis mandatory for proper management of the patients and rapid detection of contacts for quarantine purpose; real-time reverse transcriptase-polymerase chain reaction of the viral nucleic acid (RT-PCR) within body specimens mainly nasopharyngeal or oro-phangeal swab is the gold standard for diagnosis [3]. Many publications appeared mainly focusing on the diagnosis of the disease either comparing chest CT findings with RT-PCR results [4–6] or describing the disease imaging features mainly chest CT [7–9] with paucity of articles investigating the role of chest CT in disease prognosis regarding helping the physician to speed up the diagnostic work flow by properly selecting patients in need of intensive medical care, or only hospital ward care or just home quarantine. The purpose of the study was to investigate the role of chest CT findings and CT scores along with serum biomarkers namely serum ferritin and lactate dehydrogenase (LDH) in the proper effective management of COVID-19 patients.

## Methods

This retrospective study was done in the university hospital which included 250 patients [138 males 55.2%, 112 females 44.8%, age range 17–82 years, median is 49.5] from first of April till 15 June 2020. The study was approved by the hospital’s ethical committee. Inclusion criteria included patients with positive RT-PCR for COVID-19 in the nasopharyngeal or oro-phangeal swab; either having symptoms of viral pneumonia namely fever, cough, dyspnea, chest pain, or gastrointestinal symptoms mainly diarrhea and abdominal pain; or asymptomatic but with a history of contact to a positive COVID-19 patient. Exclusion criteria included any patient with chronic lung disease.

### CT scanning protocol

All the patients underwent non-enhanced chest CT examination using either 16 or 128 multislice CT scanner. The scan was taken with the patient in the supine position, head first, from above the first rib till below the diaphragm during breath holding at end of inspiration with the following parameters: tube voltage 120 kV, adaptive tube current, pitch [0.3–1.5] with slice thickness and interslice gap both of 5 mm, 0 gantry tilt, and FOV depending on the size of the patient. Images were reconstructed in both coronal and sagittal planes and were sent to a compatible PACS system.

CT images were evaluated by two experienced radiologists [more than 10 years of experience] independently and any disagreement was resolved by discussion. Axial images were reviewed along with the coronal and sagittal reformatted images on both lung window [window width 1500HU, window level 700HU] and mediastinal window [window width 350HU, window level 40HU] settings.

### Image analysis

CT images were evaluated for the following features: pulmonary manifestation localization either involving one lung or both lungs; the distribution either peripheral/subpleural, central/broncho-vascular, or both; and lesion pattern that was described in line with the Fleischner Society glossary of terms for thoracic imaging (2008) [10] including ground glass opacity [GGO] which is an area of increased attenuation not obscuring the underlying vasculature, consolidation which is an area of increased attenuation obscuring underlying pulmonary vessels, crazy paving [CP] which is an area of ground glassing with interlobular septal thickening, or mediastinal or hilar lymph nodes [with short-axis diameter more than 10 mm]. Each patient was given a severity score using a semi-quantitative method for each lobe involvement as follows: 0, no involvement; 1, < 5% involvement; 2, 5–25% involvement; 3, 26–50% involvement; 4, 51–75% involvement; and 5, > 75% involvement. The total CT score was the sum of each individual lobar score and it ranges from 0 to 25 [11]; then a severity grading was given to each patient as follows: mild if score is from 1 to 10, moderate from 11 to 19, and severe from 20 to 25.

### Laboratory markers

For each patient, serum ferritin and serum LDH were measured within 1 day of CT.

### Follow-up

A follow-up chest CT was done to 8 patients in time interval ranging from 3 to 10 days from the first scan along with serum ferritin and serum LDH due to deterioration of the clinical condition.

### Data collection

Epidemiological data, laboratory results, and CT results were obtained from the hospital information system [HIS] and image storage and transmission system [PACS].

### Statistical analysis

IBM SPSS statistics (V. 26.0, IBM Corp., USA, 2019) was used for data analysis. Data were expressed as median and percentiles for quantitative non-parametric measures and both number and percentage for categorized data.

## Results

Two hundred and fifty patients were enrolled in this study of which 138 were males [55.2%] and 112 females [44.8%], age range from 17 to 82 years with median 49.5. One hundred eighteen patients [47.2%] had underlying comorbidities as follows: forty-two [35.5%] having hypertension, 35 [29.6%] diabetes mellitus, 16 [13.5%] cardiac disease, 13 [11%] liver cirrhosis, 10 [8.4%] renal impairment, and 2 [1.7%] malignancy, one case with multiple myeloma and the other had colonic carcinoma.

A non-contrast chest CT scan was done to all patients within 1 week from the onset of symptoms or from the appearance of positive RT-PCR result, and estimation of serum ferritin level and serum LDH was done within 1 day of chest CT.

One hundred eighty-five patients [74%] were symptomatizing: 163/185 patients [88.2%] were having positive chest CT, while 22/185 patients [11.8%] had negative chest CT. Sixty-five patients [26%] were asymptomatic: 37/65 patients [56.9%] had positive chest CT whereas 28/65 patients [43.1%] showed no abnormality (Table 1).

**Table 1:** Comparison between clinical picture and chest CT findings

	Positive chest CT findings	Negative chest CT findings	Total
Symptoms	163/185, 88.2%	22/185, 11.8%	185, 74%
No symptoms	37/65, 56.9%	28/65, 43.1%	65, 26%
Total	200	50	250

Two hundred patients had positive chest CT findings and they were stratified as follows: mild [score from 1 to 10], 114 patients representing 57% (Figs. 1, 2, 3, and 4); moderate [score from 11 to 19], 65 patients [32.5%] (Figs. 5, 6, 7, 8, and 9); and severe [score from 20 to 25], 21 patients [10.5%] (Fig. 10).

**Fig. 1:**
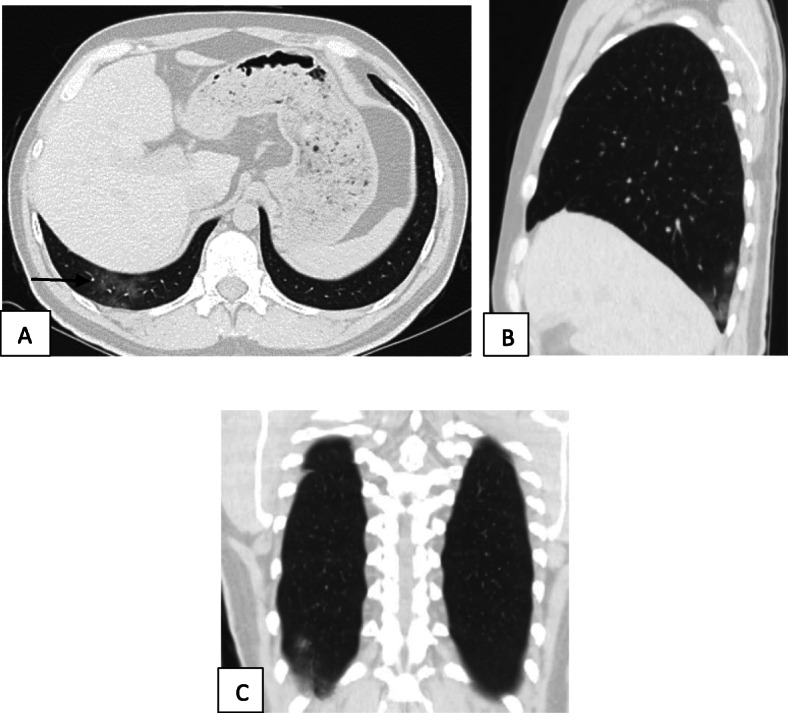
Unenhanced chest CT of a 38-year-old male patient with COVID-19. **a** An axial lung window image shows the right peripheral subpleural area of ground glass opacity [arrow]. **b** Same finding on sagittal and **c** coronal reformatted images. CT score is 1

**Fig. 2:**
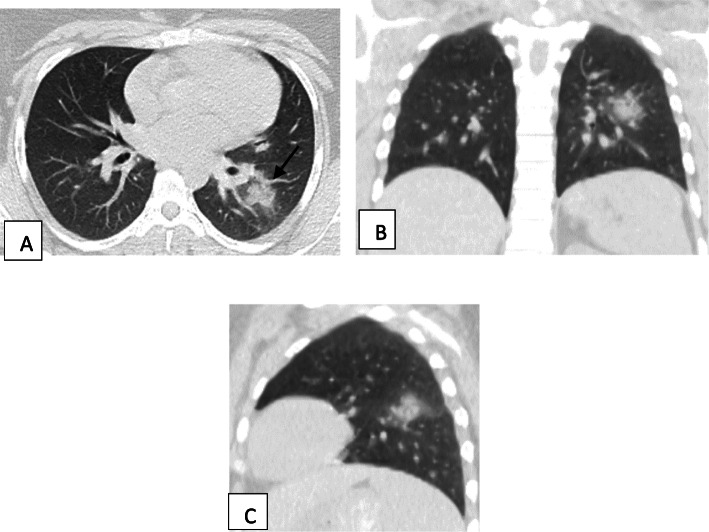
Unenhanced chest CT of a 29-year-old female patient with COVID-19. **a** An axial lung window image shows left lower lobe central broncho-vascular patch of consolidation [arrow]. **b** Same finding on the coronal and **c** sagittal reformatted images. CT score is 2

**Fig. 3:**
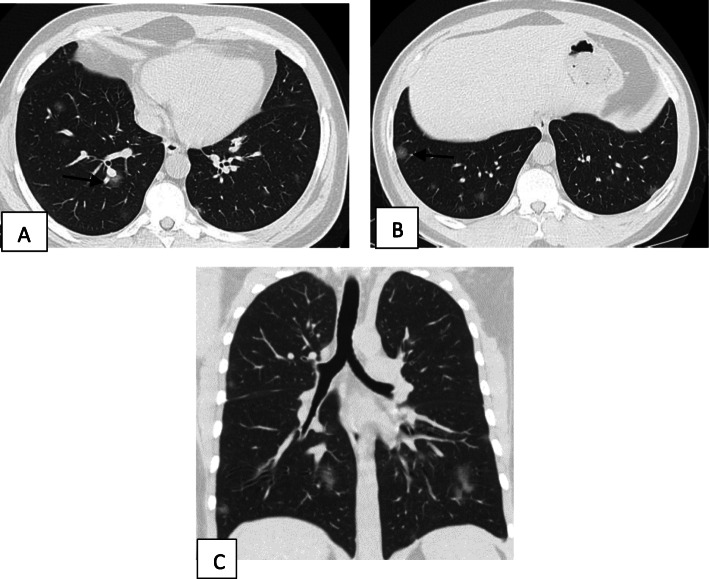
Unenhanced chest CT of a 33-year-old male patient with COVID-19. **a**, **b** Axial lung window images show bilateral subpleural and broncho-vascular areas of ground glassing opacity [arrows]. **c** Coronal reformatted images. CT score is 5

**Fig. 4:**
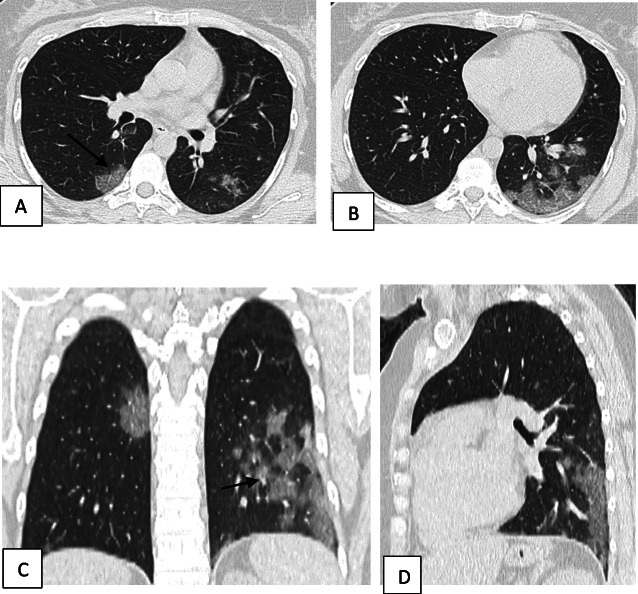
Unenhanced chest CT of a 32-year-old female patient with COVID-19. **a**, **b** Axial lung window images show bilateral subpleural and broncho-vascular areas of crazy paving [arrows]. **c** Coronal and **d** sagittal reformatted images. CT score is 7

**Fig. 5:**
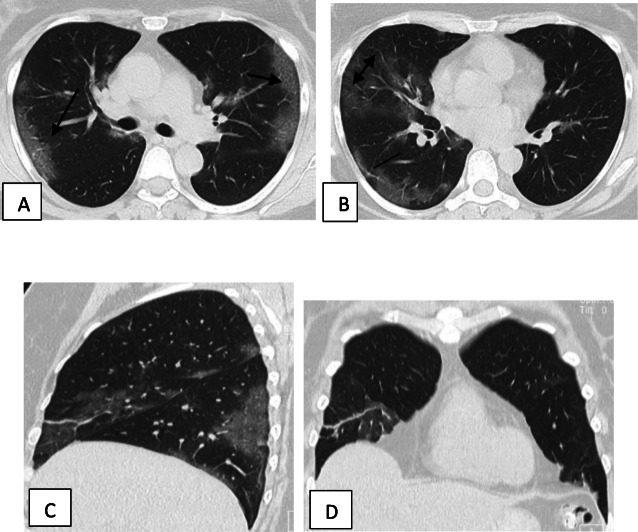
Unenhanced chest CT of a 43-year-old female patient with COVID-19. **a**, **b** Axial lung window images show bilateral subpleural areas of CP [single head arrows], subpleural and broncho-vascular areas of ground glassing opacity [double head arrow], and fibrosis [line]. **c** sagittal and **d** coronal reformatted. CT score is 11

**Fig. 6:**
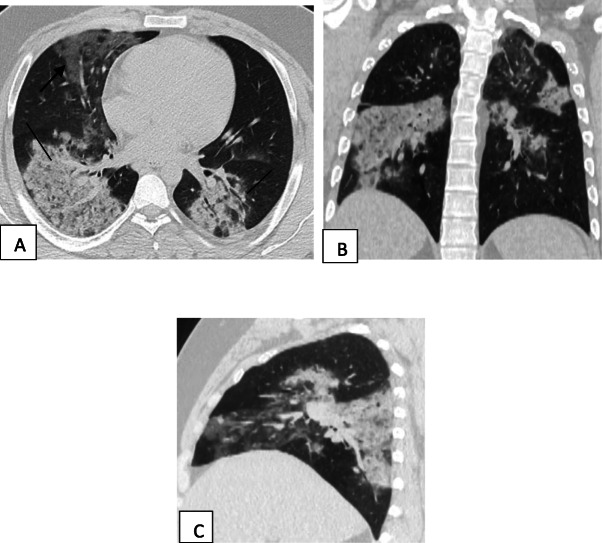
Unenhanced chest CT of a 21-year-old male patient with COVID-19. **a** An axial lung window image shows bilateral subpleural and broncho-vascular areas of ground glassing opacity [single head arrow] and crazy paving and consolidation [line]. **b** Coronal and **c** sagittal reformatted images. CT score is 18

**Fig. 7:**
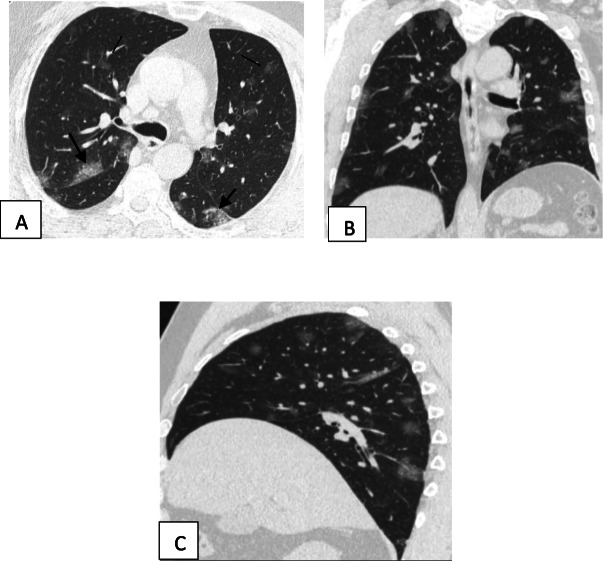
Unenhanced chest CT of a 52-year-old male patient with COVID-19. **a** An axial lung window image shows bilateral subpleural and broncho-vascular areas of ground glassing opacity [lines] and crazy paving [arrows]. **b** Coronal and **c** sagittal reformatted images. CT score is 12

**Fig. 8:**
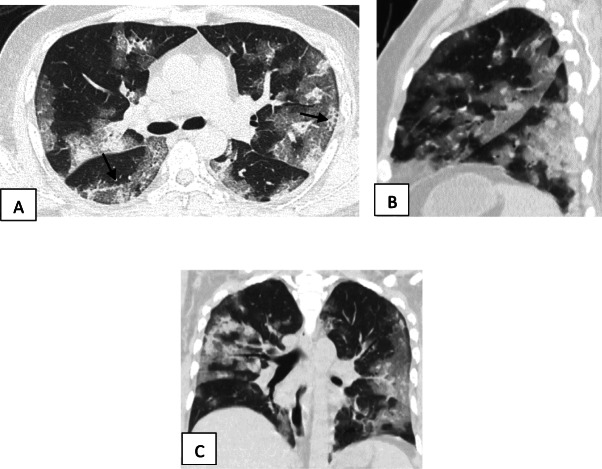
Unenhanced chest CT of 52-year-old male patient with COVID-19. **a** Axial lung window image shows bilateral subpleural and broncho-vascular areas of ground glassing opacity, consolidation, crazy paving, and fibrosis [single head arrows]. **b** Sagittal and **c** coronal reformatted images. CT score is 19

**Fig. 9:**
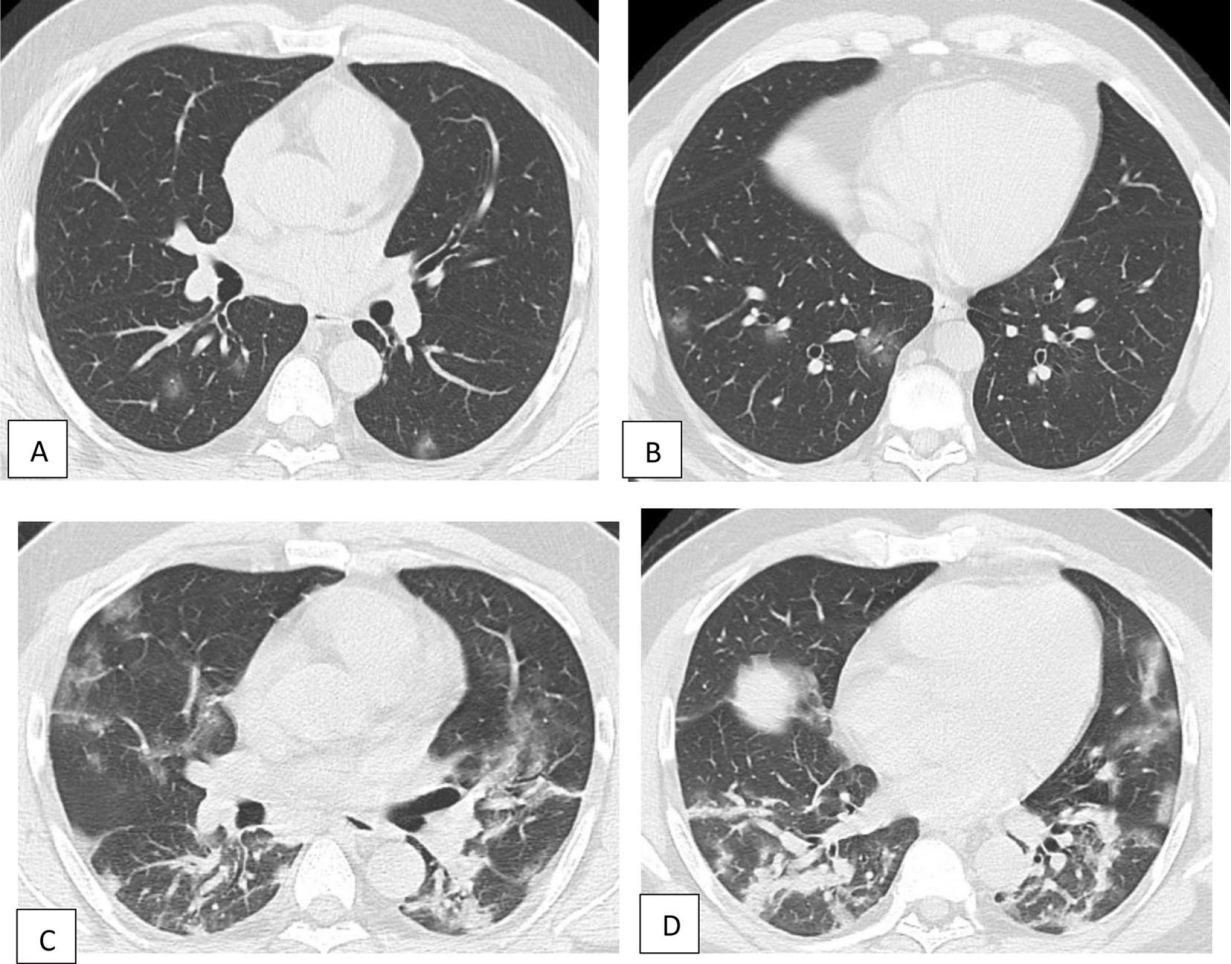
Unenhanced chest CT of a 42-year-old male patient with COVID-19. **a**, **b** Axial lung window images showing subpleural and broncho-vascular small areas of GGO with CT score 5. **c**, **d** Axial lung window images after 5 days showing the progression of the disease with increase in the number and size of the GGO areas with the development of frank areas of consolidation and fibrosis with CT score of 17

**Fig. 10:**
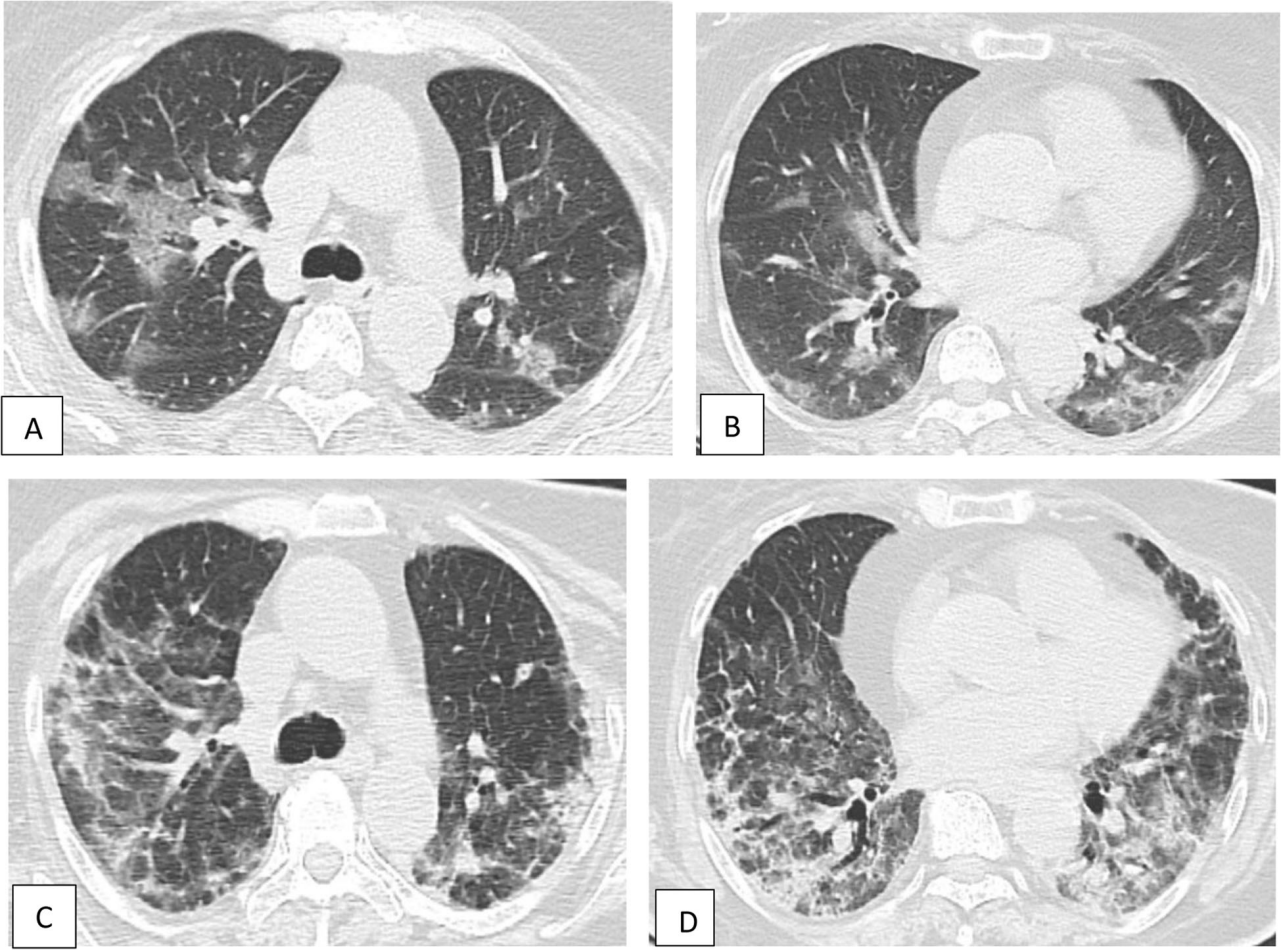
Unenhanced chest CT of a 71-year-old female patient with COVID-19. **a**, **b** Axial lung window images showing areas of ground glass opacity and crazy paving with few scattered areas of consolidation. CT score is 14. **c**, **d** Axial lung window images after 8 days showing the progression of the disease with the development of fibrosis. CT score is 20

Comparison between sex and different CT stages showed no difference in the mild stage, but in the moderate stage, females were 35.4% compared to 64.6% for males and in the severe stage, females were only 14.3% compared to 85.7% for males (Table 2); also, a comparison between age and CT scoring showed that there was a statistically significant correlation between the two factors using the Kruskal-Wallis test (Tables 3 and 4).

**Table 2:** Gender comparison between different CT stages

	Mild	Moderate	Severe	Total
**Female**	53, 46.4%	23, 35.4%	3, 14.3%	79
**Male**	61, 53.5%	42, 64.6%	18, 85.7%	121
**Total**	114, 100%	65, 100%	21, 100%	200, 100%

**Table 3:** Age comparison between different CT stages

	Mild	Moderate	Severe	Total
**17–30**	27, 23.7%	9, 13.8%	0, 0%	36
**31–50**	47 41.2%	24, 36.9%	10, 47.6%	81
**51–82**	40, 35.1%	32, 49.2%	11, 52.4%	83
**Total**	114	65	21	200

**Table 4:** Comparison between age and different CT stages [Kruskal-Wallis test]

	Number	Median	***H***	***P***	Sig
**Normal**	50	39			
**Mild**	114	42			
**Moderate**	65	50			
**Severe**	21	50			
**Total**	250				
			20.91	0	HS

Regarding laterality of positive chest CT cases, bilateral affection was 88% while unilaterality was only 12% [7.5% right lung and 4.5% left lung] (Figs. 1 and 2). Regarding the distribution of lesions, both peripheral and central were the highest percent 67% while peripheral distribution alone was 31% (Fig. 1) and central was only 2% (Fig. 2).

Regarding the pattern of the lesions, GGO was the highest pattern evident seen in 191/487 representing 39.2%, after which came consolidation in 152/487 [31.2%], then CP in 74/487 [ 15.2%] (Fig. 4), after which came fibrosis in 67/487 [13.7%] (Fig. 5), with lymph nodes seen only in three cases representing the least pattern giving only 0.6% (Table 5).

**Table 5:** Pattern of CT chest pulmonary manifestations in different CT stages

	Mild	Moderate	Severe	Total
**GGO**	105/114, 92.1%	65/65, 100%	21/21, 100%	191, 39.2%
**Consolidation**	81/114, 71%	53/65, 81.5%	18/21, 85.7%	152, 31.2%
**CP**	22/114, 19.2%	39/65, 60%	13/21, 61.9%	74, 15.2%
**Fibrosis**	21/114, 16.4%	25/65, 60%	21/21, 100%	67, 13.7%
**Lymph node**	0/114, 0%	3/65, 4.6%	0/21, 0%	3, 0.6%
**Total**				487, 100%

There was a high statistical significance between both elevated serum ferritin and serum LDH and CT staging using the Kruskal-Wallis test where ferritin was increased in 18.4% in the mild stage, 63% in the moderate, and 100% in the severe stage (Table 6) while LDH was increased in 39.4%, 92.3%, and 100% mild, moderate, and severe stages, respectively (Table 7).

**Table 6:** Comparison between elevated serum ferritin in different CT stages [normal value is between 30 and 400 ng/mL] [Kruskal-Wallis test]

	Number	Median	25Perc.	75Perc.	***H***	***P***	Sig.
**Mild**	21/114, 18.4%	492	480	557.5			
**Moderate**	41/65, 63%	634	518.5	765			
**Severe**	21/21, 100%	880	772	1264			
**Total**	83/200, 41.5%				27.451		HS

**Table 7:** Comparison between elevated serum LDH in different CT stages [normal value is between 135 and 225 U/L] [Kruskal-Wallis test]

	Number	Median	25Perc.	75Perc.	***H***	***P***	Sig.
**Mild**	45/114, 39.5%	340	237	418			
**Moderate**	60/65, 92.3%	361.5	300	450			
**Severe**	21/21, 100%	758	621.5	901			
**Total**	126/200, 63%				51.933		HS

Fifteen cases [6%] were critical cases that needed ICU admission, and all were in the severe stage while all the negative chest CT patients, mild, moderate, and the rest of the severe CT score patients 235/250 [94%] either took treatment in the ward or sent for home quarantine (Table 8).

**Table 8:** Comparison between ICU patients and ward/home patients in different CT stages

	Negative chest CT	Mild	Moderate	Severe	Total
**Ward/home**	50	114	65	6	235, 94%
**ICU**	0	0	0	15	15, 6%
**Total**	50	114	65	21	250

Regarding ICU patients, all showed severe CT scoring ranging from 21 to 23; they included one female [66 age with diabetes mellitus] and 14 male cases, with three cases that were in the age group of 31 till 50 years [one case with no comorbidity, one case with hypertension, and one with diabetes mellitus] and 11 cases that were in the age group of 51 till 82, all with comorbidity. All the patients showed bilateral lung involvement along with peripheral and central distribution. All the ICU patients showed very high levels of serum ferritin [75 percentile] reaching up to 1264, more than three times the normal range, and serum LDH [75 percentile] reaching up to 901, more than four times the normal range.

A follow-up chest CT, serum ferritin, and serum LDH were done for 8 cases [3.2%] in the interval of 3–10 days from the first chest CT due to deterioration of the clinical picture; their data were as follows: 4 cases of mild category, three of which became moderate and one remained mild but with a higher score; 3 moderate cases became severe; and one severe case remained in the severe category but with a higher score. All showed an increase in both serum ferritin and LDH compared to the first result (Table 9).

**Table 9:** Follow-up cases

1st CT score	2nd CT score	First ferritin	Second ferritin	First LDH	Second LDH
7	13	Normal	656	270	319
5	15	Normal	416	Normal	298
6	11	Normal	432	Normal	282
7	8	480	490	Normal	230
13	23	651	1232	360	620
14	20	460	580	232	742
14	21	455	232	733	505
20	23	532	1180	501	800

## Discussion

COVID-19 is a new highly contagious infectious disease that causes lower respiratory inflammation; it starts by alveolar hyaline membrane injury or interstitial edema which appears on CT as GGO then progresses to alveolar filling with exudate which is the consolidation after which an autoimmune reaction begins by activation of both humeral and cellular immunity mediated by virus-specific B and T cells causing the formation of pro-inflammatory cytokines which are the cause of interlobular septal thickening, giving rise to first crazy paving then lung fibrosis [12].

The clinical picture of the disease is diverse ranging from asymptomatic or subclinical forms to critically ill patients with acute respiratory distress syndrome [ARDS] or multi-organ failure which can occur as short as 9 days from the onset of symptoms [9], so not only diagnosis of the disease is mandatory but also monitoring the clinical course of the disease is of paramount importance to triage patients who are in need of urgent intensive medical care. Extension of pulmonary affection is easily assessed by chest CT but this is not suitable for critically ill patients, so a laboratory biomarker of disease progression is needed and here comes the role of LDH and ferritin.

LDH is an enzyme that converts lactate to pyruvate in the process of tissue breaking down, so patients with severe COVID-19 pneumonia shows an increase in the serum LDH [13]; also, ferritin is increased because it is secreted either by the macrophages that produce cytokines and account for the main immune cells present in lung parenchyma or by cytokines themselves such as interleukin-6 [14].

From 250 cases with confirmed RT-PCR for COVID-19, 185 [74%] patients were symptomatizing from which 22/185 [11.2%] showed normal chest CT; this was in agreement with Yang and Yan [15] who reported 11.8% [symptomatizing patient showing normal chest CT] while Bernheim et al. [16] showed 56%; this big difference in the percentage between the current study/Yang and Yan [15] and Bernheim et al. [16] could be explained that the latter study was done only within the first 2 days from onset of symptoms in contrary to our study and Yang and Yan that were done within 1 week.

Thirty-seven [14.8%] patients were asymptomatic and showed positive chest CT findings; this clinical-radiological discrepancy was evident in previous studies like Chung et al. [17] which showed 14% while Inui et al. [18] which reported 39.2%; this was explained by a lot of theories, like those patients were exposed to COVID-19 virus before and hence developed immunity leading to a subclinical presentation, or they are having a recent infection and in the recovery period with subsiding of symptoms at the time of chest CT done but an absence of clear clinical history of infection and lacking CT features of healing COVID-19 pneumonia weaken this theory, and the only logical explanation is that there is a time interval between chest CT manifestation and developing symptoms with the former begins first but this hypothesis needs to be more investigated [17, 18]; unfortunately, in the current study, none of those patients was followed up to see if they have developed symptoms or not.

Regarding the comparison between gender and different stages of COVID-19 pulmonary affection, there was no gender difference in the mild stage while in the moderate stage males represented 64.6% almost double of the females who were 35.4% while in the severe stage males were 85% compared to only 14.3% for females; this result was in concordance with Jin et al. [19] and Hiroki et al. [20] with the latter stating that male gender may be a predictor of a more severe form of COVID-19.

Regarding the age, there was a statistically significant correlation between older age and severity of chest CT manifestations as no patient in the severe stage was under 30 years while there was no difference between 31 and 50 years group and 51–82 years group showing 47.6% and 52.4%, respectively; this was in agreement with the US Centers for Disease Control and Prevention reporting that 8 in 10 COVID-19-related deaths have been among people aged 65 years or over [21].

Regarding the distribution of lung manifestations, unilateral lung affection was only observed in 24/200 [12%] while bilaterally was seen in 176/200 [88%], and that was in agreement with almost all previous publications [7–9, 16, 17].

Peripheral involvement was seen in 62/200 [31%] and broncho-vascular in 4/200 [2%], while both peripheral and broncho-vascular involvement was the highest percentage being 134/200 [67%]; this finding was in agreement with Wang et al. [8] who reported 56.4% both peripheral and broncho-vascular distribution. On the contrary, Shi et al. [9] reported more percentage of peripheral distribution 54% compared to 44% diffuse, and this can be explained that this study included a small number of patients, only 81 patients with some of which were asymptomatic.

Regarding the pattern of chest CT pulmonary affection in different stages, GGO was the highest percentage accounting for 191/487 [39.2%], followed by consolidation 152/487 [31.2%], then fibrosis 74/487 [15.2%], and CP 67/487 [13.7%], with lymph node only seen in 3/487 [0.6%]; these findings were in agreement with Song et al. [22].

Since the outbreak of the COVID-19 pandemic, many studies showed that the inflammatory cytokine storm is the main cause of complication of COVID-19 pneumonia leading to acute respiratory stress syndrome and multi-organ failure and even death, so laboratory detection of increased serum inflammatory markers is mandatory in order to provide those patients early proper treatment [23].

In the current study, there was a statistically high significance between increased levels of both serum ferritin and LDH in correlation with CT staging, where ferritin was increased in 18.4% in the mild stage, 63% in the moderate, and 100% in the severe stage; these findings were in agreement with Lin et al. [24] who reported that patients with severe COVID-19 pneumonia showed higher levels of serum ferritin than the non-severe patients using a multivariate logistic regression analysis showing that the serum ferritin level was an independent risk factor for disease severity.

LDH was increased in 39.4%, 92.3%, and 100% mild, moderate, and severe stages, respectively, and this was in concordance with Wu et al. [13] who reported that the higher LDH levels were found in patients with severe COVID-19. All the ICU patients showed a marked increase reaching up to more than four times the normal; these results were in agreement with the pooled study done by Henry et al. [25] who stated that increased LDH was associated with significantly increased odds of severe COVID-19 in both case-control studies and retrospective studies.

In spite that CT scoring is a subjective method for assessing the severity of COVID-19 pneumonia, it correlates well with the clinical manifestations and laboratory findings of the disease [26] helping to triage patients and reserving health care resources so only the ones with severe lung affection could be closely monitored in order to provide early aggressive treatment; in the current study, comparing the CT score for patients admitted to ICU and patients who were treated either in the hospital or at home, all the patients in ICU were in severe stage with CT scores ranging from 21 to 23, and this was in agreement with the study of 236 patients done by Colombi et al. [27] who reported a positive correlation between the extent of CT lung manifestations and intensive care unit admission or death. Also, Francone et al. [25] reported that CT score was significantly higher in critical COVID-19 patients ranging from 15 to 24 with 20.3±3 [mean value ± SD] than in the mild stage range 0–19 with 8.7±4 [mean value ± SD].

This study has limitations as none of the patients was in the pediatric age group; the study included only patients within the first week of onset of symptoms or positive RT-PCR, so late chest manifestations of the disease could not be investigated; also, there was no follow-up of cases that were asymptomatic with positive chest CT findings to find out if they developed symptoms or not or the patients that were symptomatic and with negative chest CT findings to see if they developed imaging manifestations or not, but this could be explained that the time of the study was at the peak period of the disease with a large number of cases and limited health care resources.

## Conclusion

Chest CT findings are crucial for early diagnosis of COVID-19 disease especially for asymptomatic patients for early quarantine aiming to decrease the spread of the disease. Old age and male sex are considered risk factors for poor prognosis. CT score, serum ferritin, and LDH play an important role in predicting the short-term outcome of the patients allowing prompt effective clinical monitoring and intervention to decrease both morbidity and mortality.

## Data Availability

All the data are available at the Ain-Shams University hospital information system [HIS].

## Abbreviations

RT-PCR: Real-time reverse transcriptase-polymerase chain reaction
LDH: Lactate dehydrogenase
GGO: Ground glass opacity
CP: Crazy paving

## References

[1] Guan C, Lv Z, Yan S (2020). Imaging features of coronavirus disease 2019 (COVID-19): evaluation on thin-section CT. Acad Radiol.

[2] Hani C, Trieu NH, Saab I (2020). COVID-19 pneumonia: a review of typical CT findings and differential diagnosis. Diagn Intervent Imaging.

[3] Sultan O, Altameemi H, Alghazali D (2020). Pulmonary manifestations of COVID-19: changes within 2 weeks duration from presentation. Egypt J Radiol Nucl Med.

[4] Young D, Tatarian L, Mujtaba G (2020). Chest CT versus RT-PCR for diagnostic accuracy of COVID-19 detection: a meta-analysis. J Vasc Med Surg.

[5] Ai T, Yang Z, Hou H, Zhan C, Chen C, Lv W, Tao Q, Sun Z, Xia L (2020). Correlation of chest CT and RT-PCR testing for coronavirus disease 2019 (COVID-19) in China. A report of 1014 cases. Radiology.

[6] He JL, Luo L, Luo ZD, Lyu JX, Ng MY, Shen XP, Wen Z (2020). Diagnostic performance between CT and initial real-time RTPCR for clinically suspected 2019 coronavirus disease (COVID-19) patients outside Wuhan, China. Respir Med.

[7] Sabri Y, Nassef A, Ibrahim I (2020). CT chest for COVID-19, a multicenter study-experience with 220 Egyptian patients. Egypt J Radiol Nucl Med.

[8] Wang K, Kang S, Tian R (2020). Imaging manifestations and diagnostic value of chest CT of coronavirus disease 2019 (COVID-19) in the Xiaogan area. Clin Radiol.

[9] Shi H, Han X, Jiang N, Cao Y, Alwalid O, Gu J, Fan Y, Zheng C (2020). Radiological findings from 81 patients with COVID-19 pneumonia in Wuhan, China: a descriptive study. Lancet Infect Dis.

[10] Hansell DM, Bankier AA, MacMahon H (2008). Fleischner Society: glossary of terms for thoracic imaging. Radiographics.

[11] Pan F, Ye T, Sun P, Gui S, Liang B, Li L, Zheng D, Wang J, Hesketh RL, Yang L, Zheng C (2020). Time course of lung changes on chest CT during recovery from 2019 novel coronavirus (COVID-19) pneumonia. Radiology.

[12] Li X, Geng M, Peng Y, Meng L, Lu S (2020). Molecular immune pathogenesis and diagnosis of COVID-19. J Pharm Anal.

[13] Wu M, Yao L, Wang Y, Zhu XY, Wang XF, Tang PJ, Chen C (2020). Clinical evaluation of potential usefulness of serum lactate dehydrogenase (LDH) in 2019 novel coronavirus (COVID-19) pneumonia. Respir Res.

[14] Gomez-Pasyora J, Weigand M, Kim J (2020). Hyperferritinemia in critically ill COVID-19 patients – is ferritin the product of inflammation or a pathogenic mediator?. Clin Chim Acta.

[15] Yang W, Yan F (2020). Patients with RT-PCR confirmed COVID-19 and normal chest CT. Radiology.

[16] Bernheim A, Mei X, Huang M (2020). Chest CT findings in coronavirus disease-19 (COVID-19): relationship to duration of infection. Radiology.

[17] Chung M, Bernheim A, Mei X (2020). CT imaging features of 2019 novel coronavirus (2019-nCoV). Radiology..

[18] Inui S, Fujikawa A, Jitsu M, Kunishima N, Watanabe S, Suzuki Y, Umeda S, Uwabe Y (2020). Chest CT findings in cases from the cruise ship “Diamond Princess” with coronavirus disease 2019 (COVID-19). Radiology.

[19] Jin JM, Bai P, He W (2020). Gender differences in patients with COVID-19: focus on severity and mortality. Public Health.

[20] Hiroki U, Toshiki K, Hisato T (2020). Gender difference is associated with severity of coronavirus disease 2019 infection: an insight from a meta-analysis. Crit Care Explorations.

[21] Mahase E (2020) Covid-19: Why are age and obesity risk factors for serious disease? BMJ. 10.1136/bmj.m4130 10.1136/bmj.m4130 33106243

[22] Song F, Shi N, Shan F (2020). Emerging coronavirus 2019-nCOV pneumonia. Radiology.

[23] Chen G, Wu D, Guo W, Cao Y, Huang D, Wang H, Wang T, Zhang X, Chen H, Yu H, Zhang X, Zhang M, Wu S, Song J, Chen T, Han M, Li S, Luo X, Zhao J, Ning Q (2020). Clinical and immunological features of severe and moderate coronavirus disease 2019. J Clin Invest.

[24] Lin Z, Long F, Chen X (2020). Serum ferritin as an independent risk factor for severity in COVID-19 patients. J Inf Secur.

[25] Henry BM, Aggarwal G, Wong J, Benoit S, Vikse J, Plebani M, Lippi G (2020). Lactate dehydrogenase levels predict coronavirus disease 2019 (COVID-19) severity and mortality: a pooled analysis. Am J Emerg Med.

[26] Francone M, Iafrate F, Masci G (2020). Chest CT score in COVID-19 patients: correlation with disease severity and short-term prognosis. Eur Radiol.

[27] Colombi D, Bodini FC, Petrini M (2020). Well-aerated lung on admitting chest CT to predict adverse outcome in COVID-19 pneumonia. Radiology.

